# A dermoid cyst misdiagnosed as a lipoma due to atypical magnetic resonance images: a case report

**DOI:** 10.1186/s13256-020-02584-6

**Published:** 2021-03-02

**Authors:** Taro Mikami, Chie Maeda, Fumihiko Aoki, Toshinori Iwai, Yuichiro Yabuki, Tetsuhiko Okabe, Kenichi Ohashi, Jiro Maegawa

**Affiliations:** 1 Department of Plastic and Reconstructive Surgery, Yokohama City University Graduate School of Medicine, Fukuura 3-9, Kanazawa-ku, Yokohama, Kanagawa 236-004 Japan; 2 Department of Plastic and Reconstructive Surgery, Chigasaki Municipal Hospital, Honson 5-15-1, Chigasaki, Kanagawa 253-0042 Japan; 3 Department of Plastic and Reconstructive Surgery, Fujisawa-Shounandai Hospital, Takakura 2345, Fujisawa, Kanagawa 252-0802 Japan; 4 Shonandai AOKI Plastic Surgery Clinic, Shonandai 2-10-7, Fujisawa, Kanagawa 252-0804 Japan; 5 Department of Oral and Maxillofacial Surgery, Yokohama City University Graduate School of Medicine, Yokohama, 236-004 Japan; 6 Department of Radiology, Yokohama City University School of Medicine, Fukuura 3-9, Kanazawa-ku, Yokohama, Kanagawa 236-004 Japan; 7 Department of Pathology, Yokohama City University Graduate School of Medicine, Fukuura 3-9, Kanazawa-ku, Yokohama, Kanagawa 236-004 Japan

**Keywords:** Dermoid cysts, Fat suppressed T2-weighted images, Lipoma, Misdiagnosis

## Abstract

**Background:**

Dermoid cysts are well-known lesions that manifest as subcutaneous tumors around the lateral sides of the eyebrows in young patients. Computed tomography or magnetic resonance imaging (MRI) is often performed to confirm the diagnosis. On the other hand, a lipoma is usually a circular lesion, which is sometimes observed in the upper part of the face. The signals of both T1-weighted and T2-weighted images of MRI of a lipoma are, in general, relatively highly homogenous, and the signals decrease in fat-suppressed images. Therefore, differential diagnosis between a dermoid cyst and a lipoma is usually made with MRI, especially based on fat-suppressed images. Here, we present a case of misdiagnosis of a dermoid cyst as a lipoma because of atypical magnetic resonance images.

**Case presentation:**

We report a case of a 24-year-old Japanese woman with a dermoid cyst around the lateral edge of the eyebrow. The cyst had been gradually increasing in size for the past 2 years. On MRI, it showed high internal signals on T1- and T2-weighted images. However, the signal intensity decreased homogeneously in the fat-suppressed T2-weighted images. The observed tumor had a yellowish appearance under the endoscope. On the basis of these findings, the lesion was considered a lipoma until it ruptured intraoperatively. The pathological diagnosis confirmed it to be a dermoid cyst.

**Conclusion:**

Some dermoid cysts contain lipid-rich liquid, and these may be misdiagnosed as lipomas by MRI. When a tumor is located at a common site for a dermoid cyst, the MRI images should be validated carefully if it appears like a lipoma, and the differential diagnosis should be considered carefully.

## Background

Dermoid or epidermoid cysts are congenital lesions that manifest as subcutaneous tumors around the lateral sides of the eyebrows [1]. They lead the list of differential diagnosis for tumors found in this part of the body [2]. Computed tomography (CT) is often performed to confirm the positional relationship between the tumor and the surrounding facial bone as an imaging diagnosis, whereas magnetic resonance imaging (MRI) is sometimes chosen for a qualitative diagnosis [3, 4]. A typical dermoid cyst shows isointense or hyperintense signals on T1-weighted images on MRI, while it typically shows hyperintense signals on T2-weighted images [5].

A lipoma is a space-occupying lesion. Its T1- and T2-weighted MRI images show high signal intensity. The signal intensity of the T2-weighted images usually decreases on fat-suppressed T2-weighted images uniformly. On the basis of this imaging characteristic, misdiagnosis of a dermoid cyst is considered to be rare.

This is a case report of a patient whose preoperative diagnosis was a lipoma based on the MRI images; however, the pathological findings revealed a dermoid cyst.

## Case presentation

### Onset and clinical course

A 24-year-old Japanese woman had a tumor located just above her right eyebrow. She had been aware of the lesion since she was 22 years old, and the tumor had been increasing in size without any subjective symptoms. She consulted a doctor at a clinic, as suggested by her family and friends. She had no past history of illness.

**Fig. 1.**
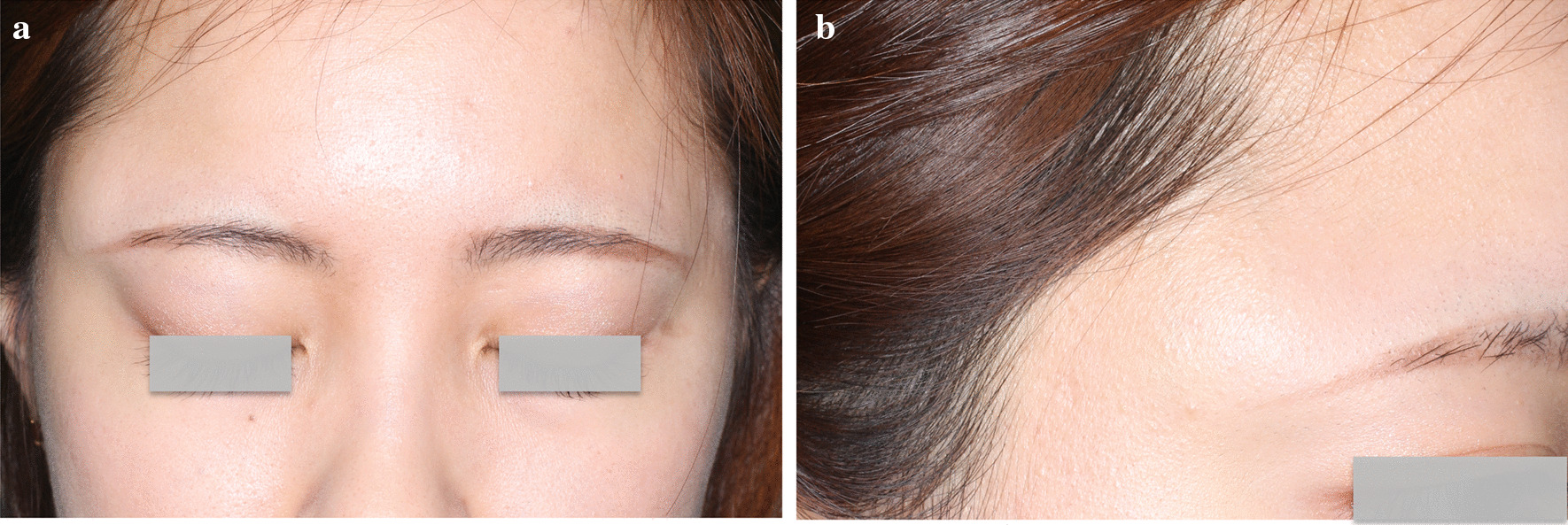
Preoperative findings of the case report. **a** Front view of the patient’s face. A dome-shaped lesion can be seen on the lateral side of the right eyebrow. **b** Lateral view of the upper part of the patient’s face. The lesion is located between the lateral end of the eyebrow and the hairline. No facial palsy was observed based on these images

The skin above the right eyebrow was slightly distended, and the diameter of the dome was about 2.5 cm. The tumor was palpable beneath the skin. There was no adhesion between the tumor and the skin; the tumor appeared to be attached to a deeper tissue layer such as the bone. The surface of the lesion was smooth, elastic, and soft without fluctuation. The protrusion became remarkable with a strong bite movement. There was no sign of facial palsy (Fig. 1).

### Diagnostic imaging

**Fig. 2.**
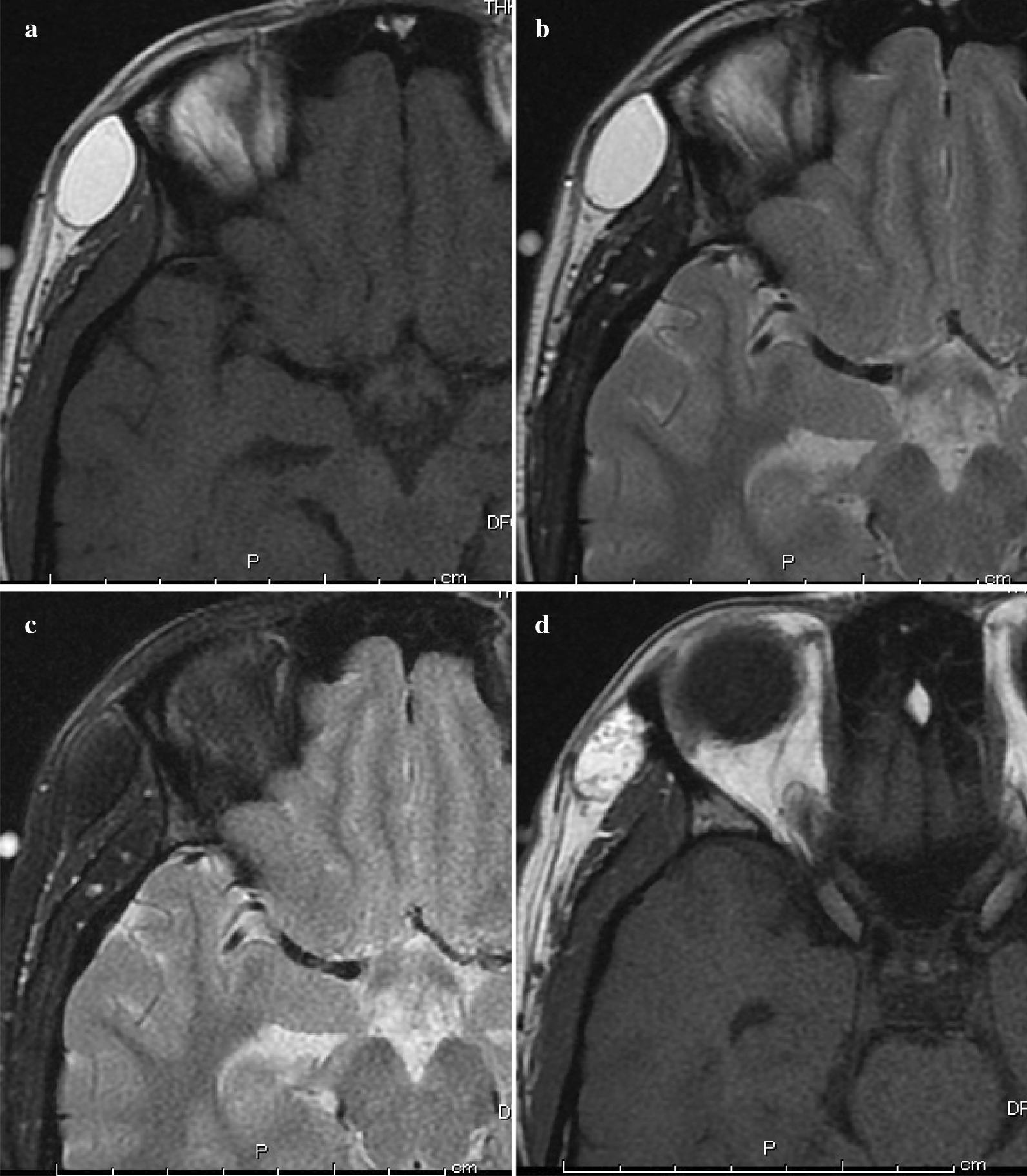
Magnetic resonance imaging before surgery. **a** T1-weighted image of the lesion. The tumor shows homogenous high intensity compared to the brain. A thin capsule is observed around the tumor. **b** T2-weighted image of the lesion. The tumor shows homogenous high intensity compared to the brain. This image also indicates a thin capsule. **c** Fat-suppressed T2-weighted image of the lesion. The internal signal from the tumor is almost completely suppressed compared to the normal T2-weighted image. **d** Another slice of the T1-weighted image of the tumor. The internal signal of the tumor is not homogenous when compared with the slice presented in **a**. This finding is thought to be atypical of a lipoma

One month after her initial consultation, she underwent MRI without any other imaging studies. The mass showed a homogenous, relatively high signal intensity compared to that of the muscle on both T1- and T2-weighted images. The actual diameter of the tumor was approximately 2.0 cm and was located between the superficial and deep temporal fascia. The signal intensity decreased homogeneously on fat-suppressed T2-weighted images; therefore, the tumor was diagnosed as a lipoma or an angiolipoma (Fig. 2).

Endoscopic surgery with an incision near the front hairline was thought to be most appropriate to preserve the esthetics; also, the tumor was quite close to the facial bone [2, 3, 6]. At that point, she was referred to our hospital for surgery, as the operation was considered more feasible under general anesthesia in order to use a nerve stimulator during surgical treatment and to perform a meticulous dissection. The surgery was performed 3 months after her first visit to our hospital.

### Intraoperative findings and clinical course

**Fig. 3.**
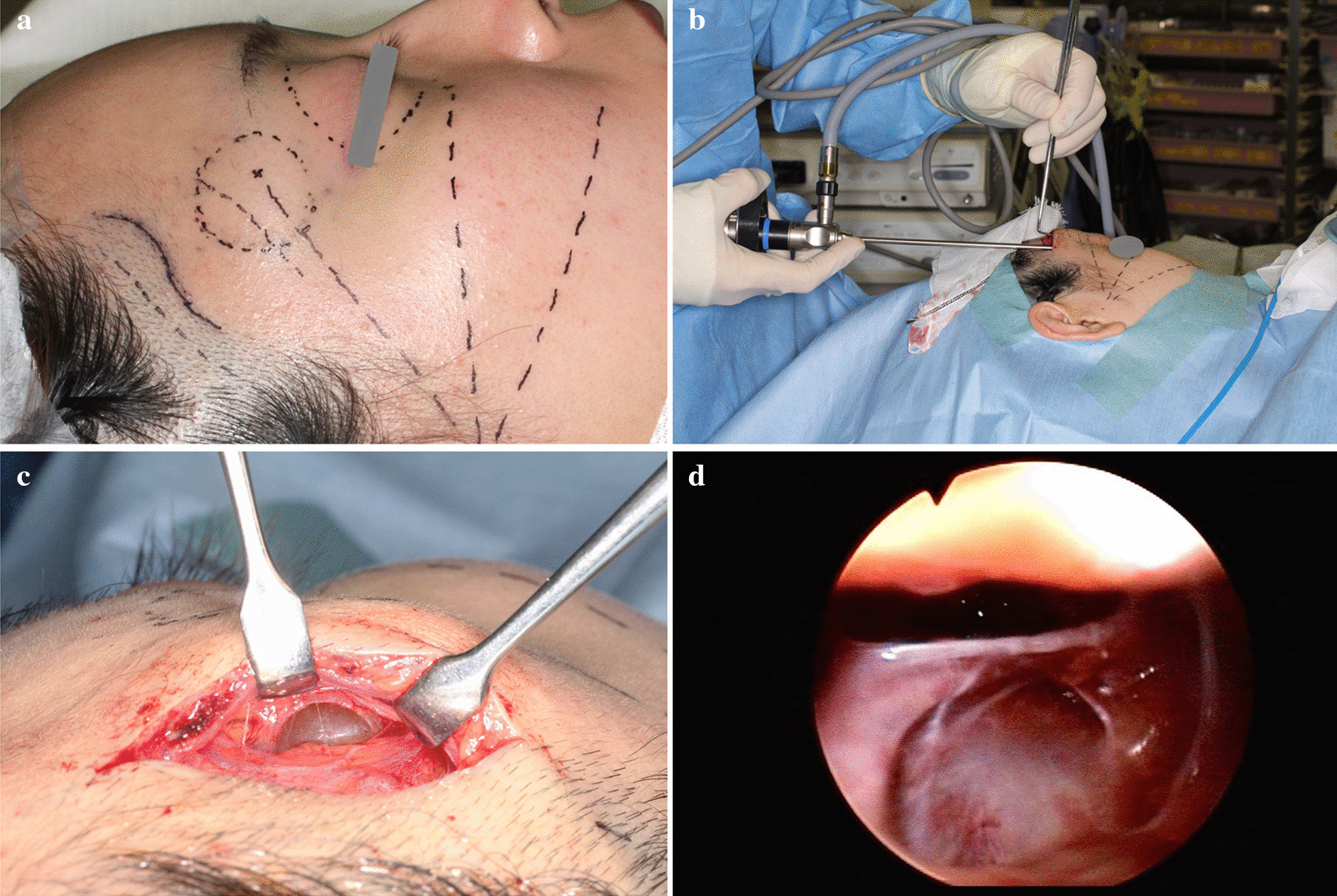
Intraoperative findings of this case report. **a** Design and markings. The orbital margin was marked with an interrupted line as a landmark. The incision line was made 1 mm behind the hairline with a lazy-S shape. The temporal branch of the superficial temporal artery, located just above the incision line, was marked with an interrupted line. The predicted lines of the temporal branch, the zygomatic branch, and the buccal branch of the facial nerve were also marked with long, interrupted lines. A small vein just above the tumor was also marked before the incision. **b** Insertion of the endoscope. The endoscope was used to check not only the tumor but also the surrounding tissues, especially fibrous tissues like nerves and blood vessels. The tip of the camera was angled at 30°. **c** Tumor appearance before rupture. The tumor could be observed directly from the incision. The color of the tumor was homogeneously yellow, like a lipoma; however, it was slightly translucent. **d** Endoscopic findings in the cavity after removal of the tumor. A circular cavity was observed endoscopically. The cavity was considered to be surrounded by the bone covered by the periosteum. There was no fibrous tissue (such as nerve fibers) in this view

An incision was created at her front hairline followed by dissection with the assistance of an endoscope (Fig. 3a, b). Blunt dissection was performed just above the deep temporal fascia to the caudal side with nerve hooks, and the tumor was found in the fat tissue. There was no movement of the frontalis muscle during these procedures, although a nerve stimulator with a 2-mA current was applied to the fibrous tissues that appeared to be nerves.

The tumor was dark yellow and capsulated by thin fibrous tissue, making its identification easy in the surrounding fatty tissue (Fig. 3c). While blunt dissection between the tumor and the surrounding tissue was performed with the endoscope, yellow and clear serous liquid suddenly drained from the tumor. After shrinkage of the tumor, the tumor capsule was separated from the surrounding tissue with sharp dissection. The periosteum of the upper margin of the orbit was considered to be the farthest margin of the lesion (Fig. 3d). A strip of silicon drain was left on the periosteum through the incised portion to prevent hematoma formation in the dissected space. The wound was closed layer by layer with absorbable and non-absorbable strings. The right frontalis muscle did not move immediately after general anesthesia was discontinued.

### Clinical course after surgery

**Fig. 4.**
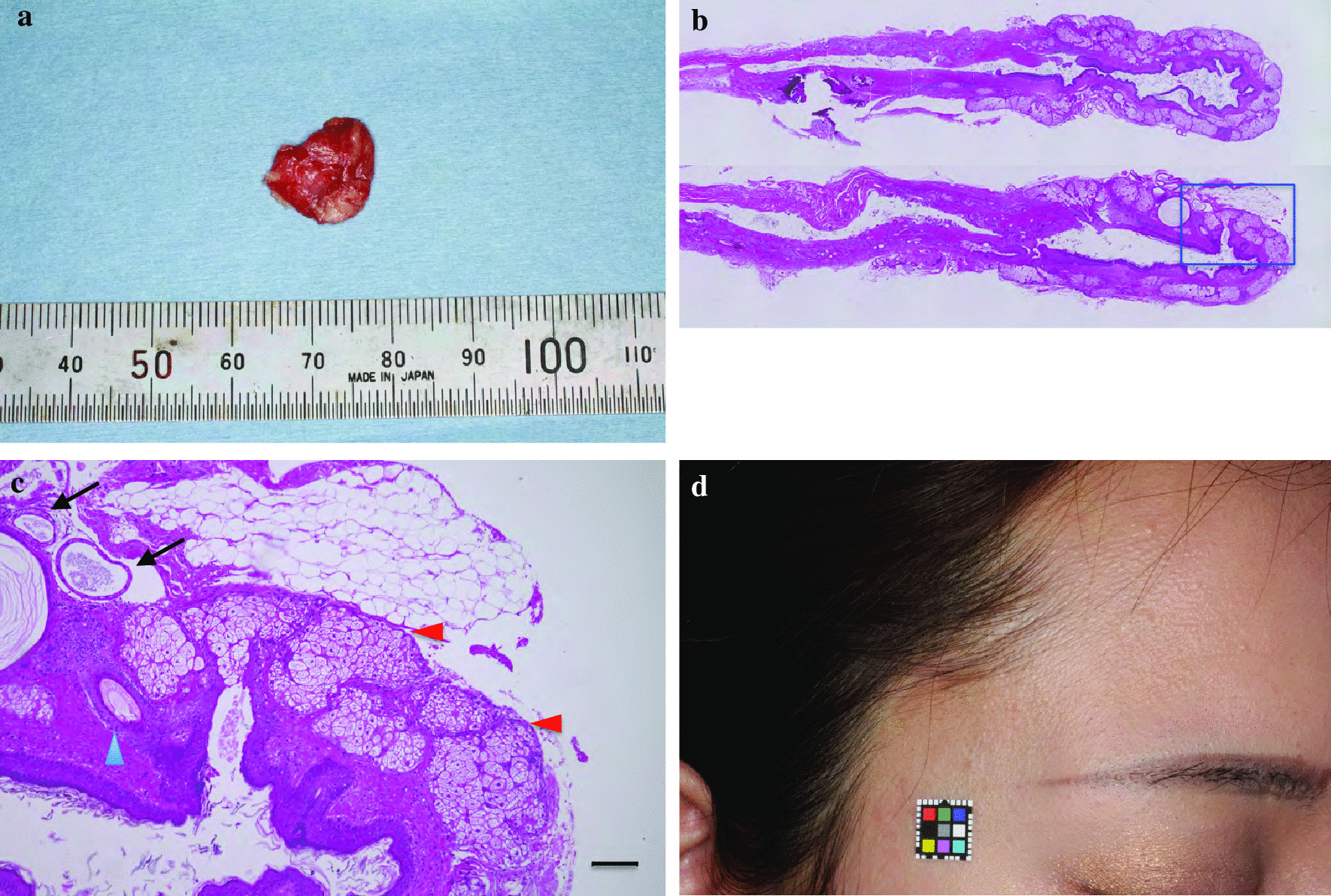
Pathological findings and postoperative scar. **a** Appearance of the resected tumor. As the tumor ruptured during surgery and almost all of the content was liquid, the resected lesion shrank; otherwise, it would have been extirpated en bloc. **b** Microscopic findings of the tumor in a low-power field. Two specimens are presented with a hematoxylin–eosin stain (HE stain). The inset with blue lines in the lower specimen is shown in **c**. **c** Microscopic findings of the tumor in a high-power field. The lumen of the tumor is covered with stratified cells with hair follicles (blue arrowhead). Many sebaceous glands are observed just below the stratified cells (red arrowheads). There are some daughter lesions in this specimen (black arrows). The scale bar is 100 μm. **d** Operative scar 1 year after surgery. The scar is inconspicuous as it is located just behind the hairline. The patient was aware and accepted that a scar would be left as a result of the surgery

The clinical course after surgery was uneventful. The function of the right frontalis muscle recovered completely the day after the operation as expected. The pathological diagnosis was a dermoid cyst (Fig. 4ac, Additional file 1: Data S1). No adverse symptoms have been observed in the year following the surgery. There was no evidence of recurrence on the follow-up MRI performed 1 year after the surgery (Additional file 2: Data S2a, Additional file 3: Data S2b). The operative scar was inconspicuous 1 year after the surgery (Fig. 4d, Additional file 4: Data S3).

## Discussion and conclusions

This case report has two important “takeaway” lessons. Firstly, a tumor found just above the eyebrow should have a dermoid cyst in the differential diagnosis, as this area is a common site. The second one is that few cases of dermoid cysts show lipoma-like images on MRI. Therefore, details of the images such as the tumor capsule and the internal signals should have been considered [5]. Tumors just above the eyebrow in the young are suspected to be dermoid or epidermoid cysts in most cases [1]. However, surgeons usually perform operations in cases where preoperative MRI images show the typical pattern of a lipoma, especially when the internal signals are suppressed uniformly on fat suppressed T2-weighted images. In this case, we considered the tumor to be a lipoma until the yellow fluid drained out because the endoscopic findings revealed a round, yellow tumor.

Histopathological findings revealed many sebaceous glands in the subepithelial layers of the specimens. From these findings, it was inferred that these glands secreted the liquid found in the tumor, which was thought to be lipid rich. In retrospect, the preoperative MRI images of this case revealed that the tumor was surrounded by a thin capsule, and some T1-weighted images showed a heterogeneous internal signal that was not a typical pattern of a lipoma (Fig. 2a, b, d).

Dermoid cysts are observed in intracranial and intraperitoneal spaces except around the orbit [4, 7–10]. Therefore, surgeons could misinterpret the findings and approach a dermoid cyst, which contains lipid-rich fluid (as seen in the present case), as a lipoma regardless of the use of endoscopes. The aim of this case report is to draw the attention of surgeons to similar presentations in other patients.

In conclusion, we report a case of a dermoid cyst around the lateral eyebrow that was misdiagnosed as a lipoma based on the preoperative MRI findings. Imaging diagnosis should be examined closely not only regarding the major part of the lesion but also regarding the details such as the capsule and the irregular findings in the smaller areas. Common sites for some lesions should be considered in the differential diagnosis, because some cases have atypical images that do not correspond with their pathological findings. In such cases, other imaging modalities and diagnoses should be considered to avoid misdiagnosis or misinterpretation of the MRI findings.

## Supplementary information

**Additional file 1: Data S1.** Microscopic findings of the tumor in the high-power field in other parts of the specimens. a. Magnified findings of the left inlet. Most of the lumen has lost the stratified inner layer. The blue arrowhead shows the keratins that may have reflected the irregular images seen on the MRI before surgery as seen in Fig. 2d. b. Magnified findings of the right inlet. The lumen contains short fragments of hair, indicated by red arrowheads in this image. This specimen also contains daughter lesions indicated by the yellow arrow as seen in Fig. 4c.

**Additional file 2: Data S2a.** A cine movie of T2-weighted images around the right orbit 1 year after surgery. The exact position of the tumor was marked on the skin. There is no sign of recurrence.

**Additional file 3: Data S2b.** A cine movie of T1-weighted images around the right orbit 1 year after surgery. This movie also shows no signs of recurrence.

**Additional file 4: Data S3.** Frontal views of the patient before and after the surgery. The image on the top is the same image as Fig. 1a. The image on the bottom shows the status 1 year after the surgery. A slight concavity is observed just behind the lateral edge of the right eyebrow.

## Data Availability

The data are available from the corresponding author upon reasonable request.

## Abbreviations

CT: Computed tomography
MRI: Magnetic resonance imaging
