# Supplementary figures and images for: A dermoid cyst misdiagnosed as a lipoma due to atypical magnetic resonance images: a case report
Source: J Med Case Rep. 2021 Mar 2;15:99. doi: 10.1186/s13256-020-02584-6 (PMC7923823; doi:10.1186/s13256-020-02584-6)

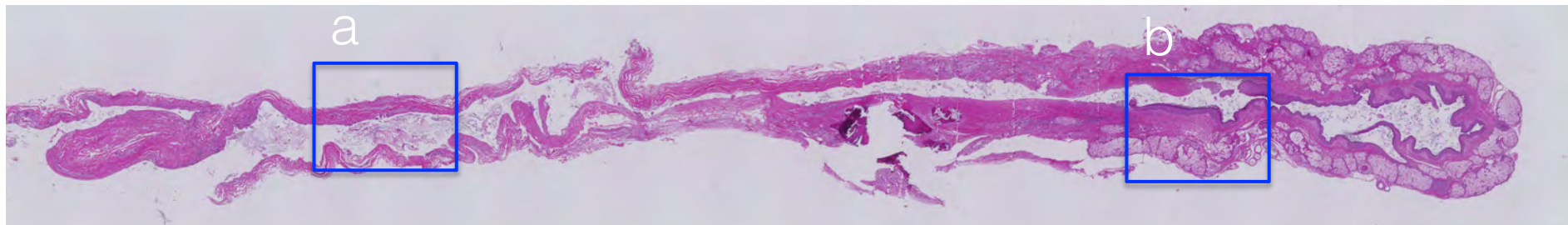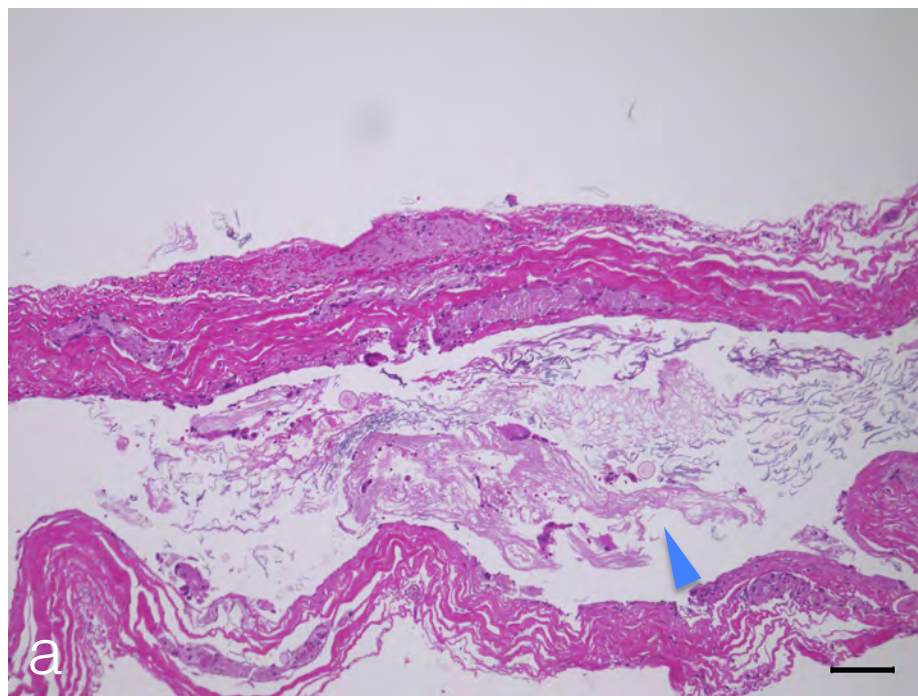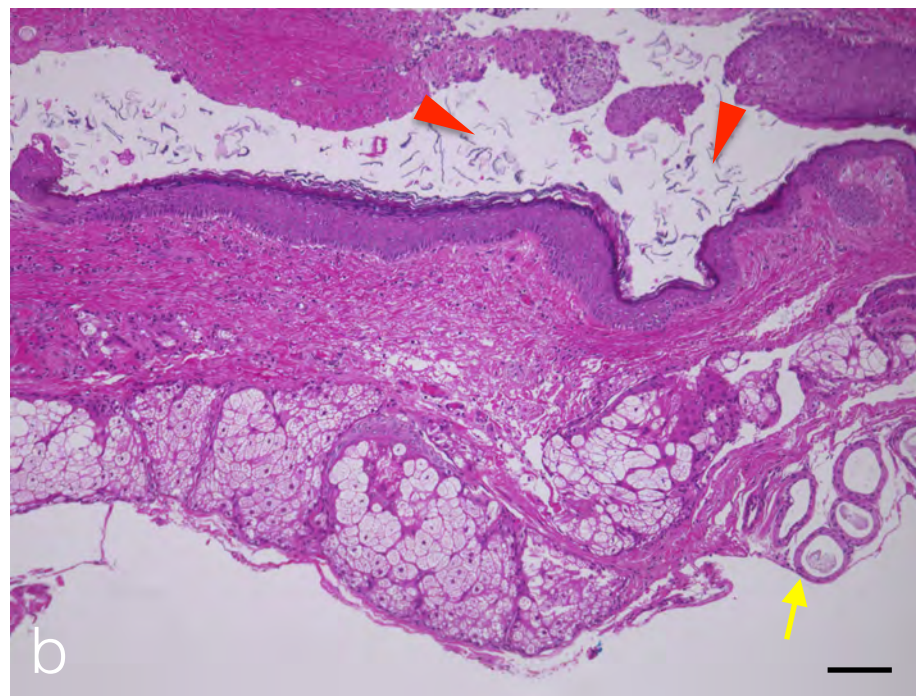

Supplement: Supplementary file 1 — Additional file 1: Data S1. Microscopic findings of the tumor in the high-power field in other parts of the specimens. a. Magnified findings of the left inlet. Most of the lumen has lost the stratified inner layer. The blue arrowhead shows the keratins that may have reflected the irregular images seen on the MRI before surgery as seen in Fig. 2d. b. Magnified findings of the right inlet. The lumen contains short fragments of hair, indicated by red arrowheads in this image. This specimen also contains daughter lesions indicated by the yellow arrow as seen in Fig. 4c. [file 13256_2020_2584_MOESM1_ESM.pdf]

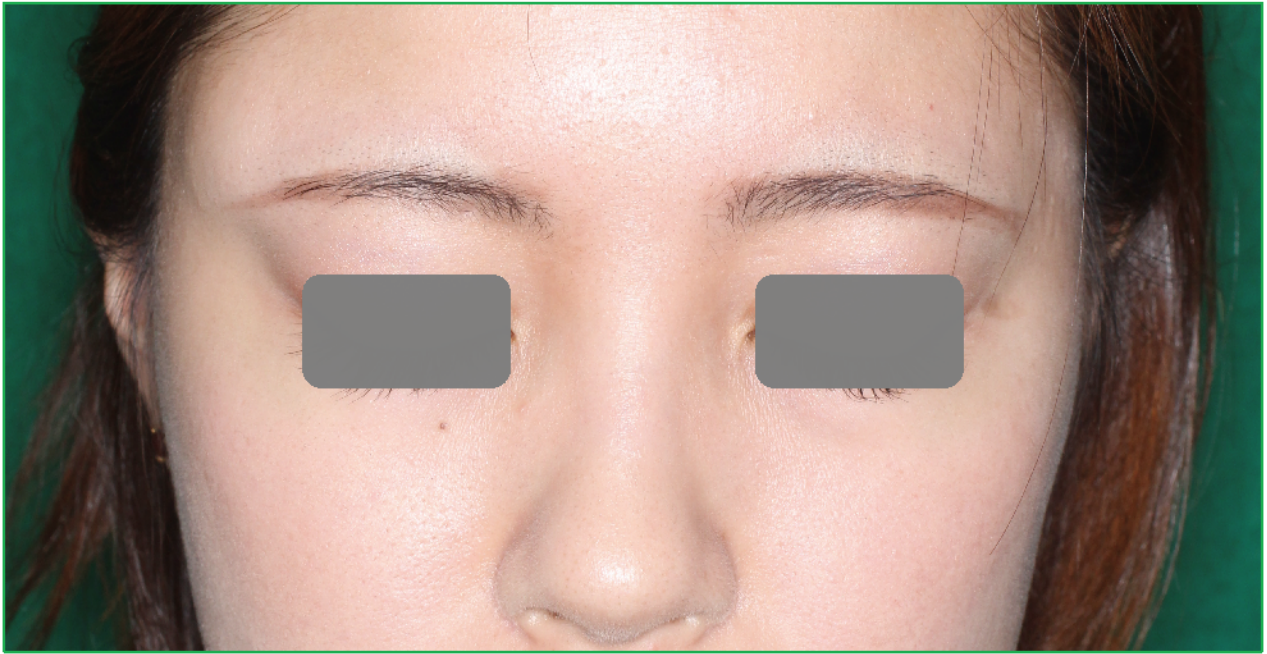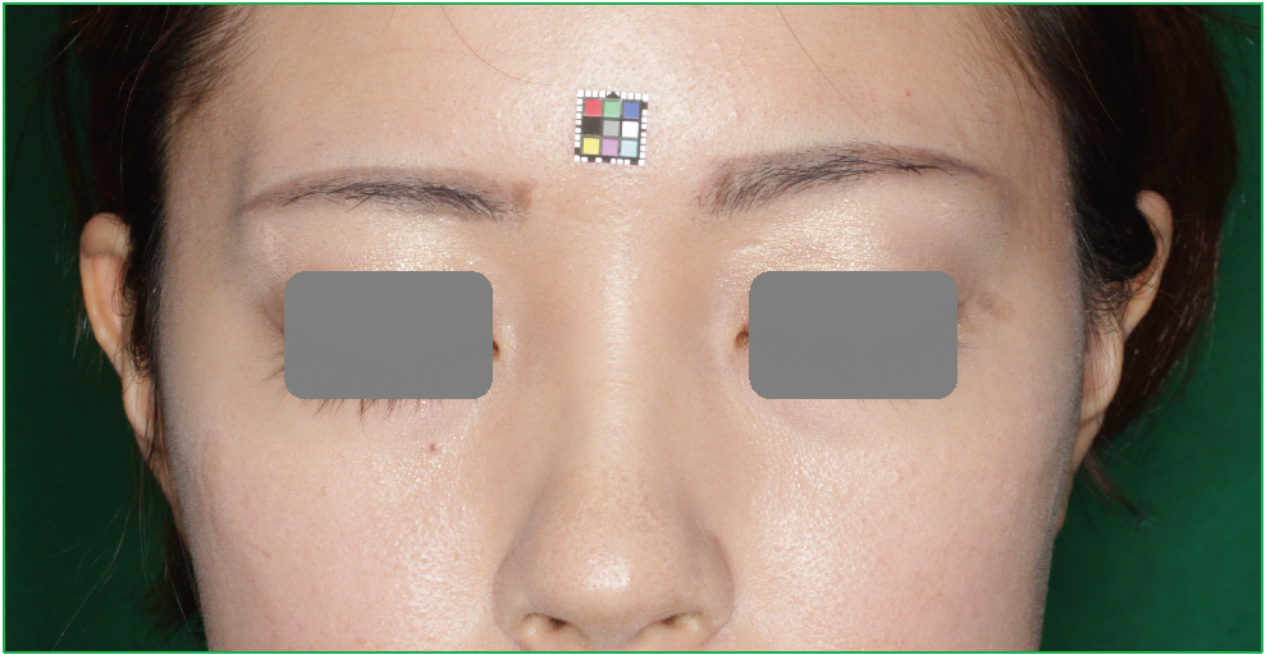

Supplement: Supplementary file 4 — Additional file 4: Data S3. Frontal views of the patient before and after the surgery. The image on the top is the same image as Fig. 1a. The image on the bottom shows the status 1 year after the surgery. A slight concavity is observed just behind the lateral edge of the right eyebrow. [file 13256_2020_2584_MOESM4_ESM.pdf]
